# Assessment of the relationship between smoking and meniscal injury

**DOI:** 10.1590/0100-3984.2023.0081

**Published:** 2023

**Authors:** Mehmet Şirik, Duygu İmre Yetkin, İbrahim İnan

**Affiliations:** 1 Department of Radiology, Faculty of Medicine, Adıyaman University, Adıyaman, Turkey; 2 King’s College Hospital, Dubai, United Arab Emirates

**Keywords:** Tobacco products, Smoking/adverse effects, Knee joint, Meniscus/injuries, Produtos do tabaco, Fumar/efeitos adversos, Articulação do joelho, Menisco/lesões

## Abstract

**Objective:**

To determine whether being a smoker and the years of smoking correlate with the presence and degree of meniscal injury.

**Materials and Methods:**

Individuals who underwent magnetic resonance imaging of the knee were divided into two groups: smokers and nonsmokers. For each smoker, the total smoking history was calculated by multiplying the daily consumption (packs/day) by the years of smoking, and the result is expressed as pack-years. In the evaluation of meniscal injury, the grade of injury was recorded. The thickness of the subcutaneous adipose tissue, as an indicator of obesity, was measured at the medial knee on axial plane images. The relationships that smoking and obesity had with meniscal injury were analyzed statistically.

**Results:**

A total of 156 individuals were included in the study. The smoker group consisted of 48 individuals (30.8%), and the nonsmoker group consisted of 108 (69.2%). The meniscus was normal in one (2.1%) of the smokers and in 32 (29.6%) of the nonsmokers (*p* < 0.0001). The median subcutaneous adipose tissue thickness was 23 mm and 24 mm in the smokers and nonsmokers, respectively (*p* = 0.900). A moderate but statistically significant correlation was observed between packs/day and injury grade, as well as between pack-years and injury grade (r = 0.462, *p* = 0.001 and r = 0.523, *p* = 0.001, respectively). Smoking and age significantly increased the risk of meniscal injury, by 31.221 times (*p* = 0.001) and 1.076 times (*p* < 0.001), respectively.

**Conclusion:**

Our findings indicate that current smoking and smoking history correlate significantly with meniscal injury grade.

## Abstract (pt)

**Objetivo:**

Determinar a correlação do tabagismo e do tempo de tabagismo com a presença e o grau de lesão do menisco.

**Materiais e Métodos:**

Foram incluídos pacientes submetidos a ressonância magnética do joelho e divididos em dois grupos: fumantes e não fumantes. O consumo total de cigarros dos pacientes foi calculado multiplicando-se a quantidade diária de cigarros (maços/dia) pelo tempo de tabagismo (anos). O grau mais alto de lesão foi registrado na avaliação da lesão meniscal. A espessura do tecido adiposo subcutâneo foi medida com base em imagem do joelho em plano axial, como indicador de obesidade. A relação entre tabagismo, obesidade e lesão meniscal foi analisada estatisticamente.

**Resultados:**

Foram incluídos no estudo 156 indivíduos. O grupo de fumantes consistiu de 48 (30,8%) indivíduos e o grupo de não fumantes, de 108 (69,2%) indivíduos. O menisco estava normal em um (2,1%) fumante e em 32 (29,6%) não fumantes (*p* < 0,0001). A espessura média do tecido adiposo subcutâneo em fumantes foi 23 mm e nos não fumantes foi 24 mm, com valor de *p* = 0,900. Foi observada correlação moderada e estatisticamente significante entre o grau de lesão por pacote/dia (r = 0,462, *p* = 0,001) e o grau de lesão por pacote × ano (r = 0,523, *p* = 0,001). O tabagismo e a idade afetaram significativamente o risco de lesão do menisco (31,221 vezes, *p* = 0,001 e 1,076 vez, *p* < 0,001, respectivamente).

**Conclusão:**

Foi encontrada correlação significativa entre tabagismo/dia e grau de lesão do menisco e pacote de tabagismo × ano e grau de lesão do menisco.

## INTRODUCTION

The importance of the meniscus for the normal functioning of the knee joint and the sustainability of the joint function is known. The meniscus is an important structure that ensures the durability of the femorotibial joint, the distribution of the load in the joint, lubrication for motion, and nourishment for the joint^(1)^. Meniscal injuries are common with degenerative meniscal changes, and the rate of such injuries is reported to be between 11.1% and 31.5% in asymptomatic individuals^(2)^. Arthroscopic partial meniscectomy after meniscal injury has been reported to be the most common orthopedic surgical intervention in the United States^(3)^. That highlights the importance of meniscal injury in terms of lost workplace productivity and health care expenditures. Although the importance of meniscal injury is known, less is known about its epidemiology. In previous studies, risk factors for meniscal injury have been described^(3)^. One such risk factor is obesity, which increases bone density in subchondral areas in the joint region. The greater load on the cartilage creates a mechanism for injury in the structure of the meniscus^(2)^. Despite many studies investigating the relationship between smoking and musculoskeletal pathologies, this issue remains unclear because of contradictory results. Some studies have indicated that smoking has a protective effect on these pathologies^(4-7)^, whereas others indicate that smoking increases the risk of meniscus injury^(8-12)^. Smoking is known to be associated with deterioration and delay in wound healing after many orthopedic operations, including those for the repair of fractures, joint cartilage lesions, and meniscal injuries. However, to our knowledge, there have been no studies investigating the relationship between meniscal injury and smoking. Our hypothesis was that smoking increases the risk of meniscal injury independent of subcutaneous adipose tissue thickness, age, and sex.

The aim of this study was to determine whether smoking correlates with the incidence or grade of meniscal injury, as well as to demonstrate the effect of age, sex, and subcutaneous adipose tissue thickness on meniscal injury. If smoking can be shown to be an etiological factor for meniscal injury, the morbidity and financial burden associated with such injury can be reduced by promoting smoking cessation.

## MATERIALS AND METHODS

This was a prospective study, conducted between 15 November 2018 and 15 June 2019, of individuals who underwent magnetic resonance imaging (MRI) of the knee at our clinic. The study was approved by the local ethics committee (Reference no. 2018/7-19), and all study participants gave written informed consent. Only individuals ≥ 18 years of age were included in the study. Individuals who had experienced knee trauma or had undergone knee surgery were excluded. Because participation in sports puts high loads on the meniscus^(13)^, thus increasing the risk for meniscal injury^(14)^, individuals with a known history of regularly engaging in sports were excluded from the study. The participants were divided into two groups: smokers and nonsmokers. Daily cigarette consumption (packs/day) and years of smoking were recorded for the individuals in the smoker group. For each participant, the total smoking history was calculated by multiplying the number of packs per day by the number of years of smoking, resulting in the number of pack-years. The radiologists who evaluated the MRI scans were blinded to the clinical data, such as the smoking status, age, and sex of the participants.

### Technical parameters

All MRI examinations were performed in a 1.5-T scanner (Achieva; Philips Medical Systems, Best, The Netherlands) with a standard protocol for knee examination consisting of four sequences: three proton density-weighted spectral attenuated inversion recovery sequences (in the sagittal, coronal, and axial planes, respectively); and one coronal T1-weighted turbo spin-echo sequence. The parameters for the spectral attenuated inversion recovery sequences were as follows: sagittal plane-repetition time (TR) of 3034 ms, echo time (TE) of 30 ms, slice thickness of 3.5 mm, and interslice gap of 0.3 mm; coronal plane-TR of 3034 ms, TE of 30 ms, slice thickness of 3.5 mm, and interslice gap of 0.3 mm; and axial plane-TR of 3034 ms, TE of 30 ms, slice thickness of 3.5 mm, and interslice gap of 0.3 mm. For the T1-weighted turbo spin-echo sequence, the following parameters were used: TR of 560 ms, TE of 17 ms, slice thickness of 3.5 mm, and interslice gap of 0.6 mm.

### Image evaluation

**Figure 1:**
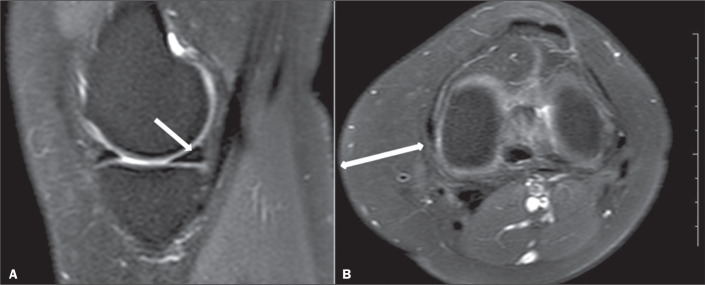
A 28-year-old nonsmoker. A: Grade 1 injury of the medial meniscus is shown as a focal area of hyperintensity in the single slice (arrow). B: The thickness of the subcutaneous adipose tissue was measured in the medial knee at the level of the joint space (arrow).

**Figure 2:**
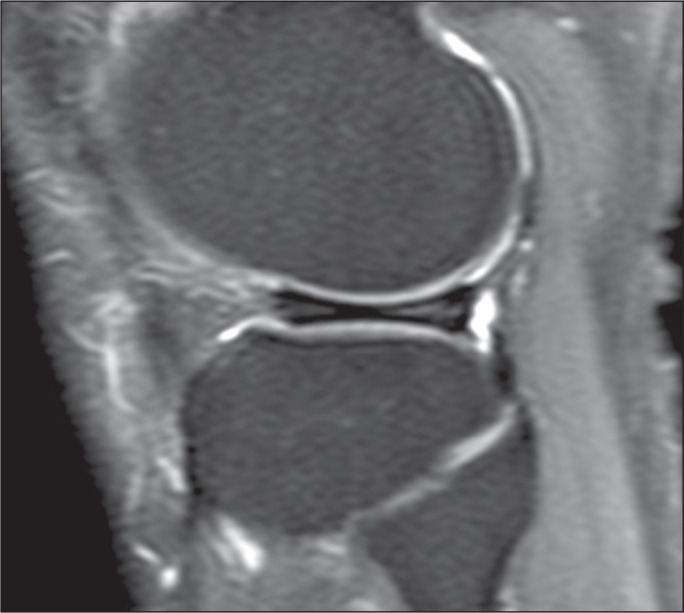
Grade 2 injury to the body of the medial meniscus in a 33-year-old patient with a 12 pack-year history of smoking.

**Figure 3:**
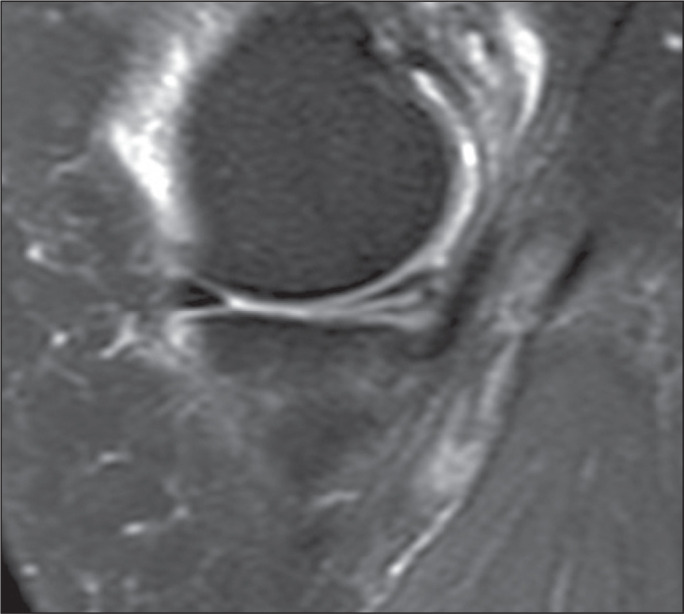
Grade 3 injury to the posterior horn of the medial meniscus in a 38-year-old patient with an 11 pack-year history of smoking.

Two radiologists, with 8 and 25 years of experience, respectively, evaluated the MRI scans by establishing consensus. The severity of each meniscal injury was evaluated. Grade 0 was indicative of a normal meniscus (a negative case). Grade 1 was characterized by a small focal area of hyperintensity not reaching the surface of the meniscus (Figure 1). Grade 2 was characterized by a linear hyperintensity not reaching the surface of the meniscus (Figure 2). Grade 3 was characterized by abnormal meniscal hyperintensities extending to at least one surface; that is, a meniscal tear^(15)^, as illustrated in Figure 3. Grade 1, 2, and 3 injuries were classified as positive cases. In the assessment of the meniscal injury, the grade was recorded. If both knees were assessed, the highest grade was recorded. To provide a standardized indicator of obesity, the thickness of the subcutaneous adipose tissue was measured in the medial knee on axial plane images, at the same level (at the level of the joint space) in all participants (Figure 1). The relationship between smoking, obesity, and meniscal injury was analyzed statistically.

### Statistical analysis

Statistical analyses were performed with the IBM SPSS Statistics software package, version 21.0 (IBM Corporation, Armonk, NY, USA). Results are presented as absolute value and percentage, as mean ± standard deviation, or as median and range. Categorical variables were analyzed by using Pearson’s chi-square test and Fisher’s exact test. Conformity of continuous variables to normal distribution was examined with visual methods (histograms and probability graphs) and analytical methods (Kolmogorov-Smirnov and Shapiro-Wilk tests). In the comparisons of continuous variables between the two groups, Mann Whitney U tests or t-tests for independent groups were used, according to the conformity to normal distribution. Correlation coefficients and statistical significance for the relationships between variables, at least one of which was not normally distributed or ordinal, were calculated with Spearman’s test. The point-biserial correlation coefficient was also calculated for continuous and qualitative data. The effects of different predictors determined by pairwise comparisons in predicting the presence of meniscal injury were evaluated by logistic regression analysis. Sex, age, smoking status, and subcutaneous adipose tissue thickness, which were determined as a result of univariate analyses (*p* < 0.25) and have rarely been investigated according to the literature, were taken as independent variables, whereas the presence and absence of meniscal injury were taken as dependent variables. The model created for multivariate analysis was considered valid because the omnibus test result was *p* < 0.001 and the Hosmer-Lemeshow test result was *p* = 0.981. The Nagelkerke R^2^ for the model was 33.6%, and the overall discrimination of the model was 82.1%. In the logistic regression, the enter method was used and the level of statistical significance was set at *p* < 0.05.

## RESULTS

A total of 26 individuals were excluded: six because they practiced sports regularly; 10 because they had previously undergone knee surgery; and 10 because the images acquired were not of diagnostic quality. Therefore, the final sample comprised 156 participants. Of those, 71 (45.5%) were male and 85 (54.5%) were female. Ages ranged from 19 to 70 years, and the median age was 37.5 years. Of the 156 participants, 48 (30.8%) were smokers and 108 (69.2%) were nonsmokers. Meniscal injury was identified in 123 individuals (78.8%). Of those 123 meniscal injuries, 30 (19.2%) were classified as grade 1, 62 (39.7%) were classified as grade 2, and 31 (19.9%) were classified as grade 3. In the smoker group, the time since the start of smoking ranged from 1 year to 50 years, with a median of 12.5 years. The minimum total smoking history ranged from 1 pack-year to 64 pack-years, with a median of 10 pack-years. For subcutaneous adipose tissue thickness measured at the knee level, the minimum was 10 mm, the maximum was 42 mm, and the median was 19 mm. The presence of meniscal injury did not differ significantly between men and women (*p* = 0.688). As shown in Table 1, meniscal injury was significantly more common among the smokers than among the nonsmokers (*p* < 0.0001). When age, years of smoking, daily cigarette consumption, total smoking history, and subcutaneous adipose tissue thickness were compared with the presence of meniscal injury, the *p*-values calculated were 0.001, 0.125, 0.833, 0.167, and 0.900, respectively (Table 2).

**Table 1:** Comparison between individuals with and without meniscal injury, by sex and smoking status.

Variable	Meniscal injury		*P*
	Positive (n = 123)	Negative (n = 33)	
Sex, n (%)			0.688
Male	57 (80.3)	14 (19.7)	
Female	66 (77.6)	19 (22.4)	
Smoking, n (%)			< 0.0001
Yes	47 (97.9)	1 (2.1)	
No	76 (70.4)	32 (29.6)	

**Table 2:** Comparison between individuals with and without meniscal injury, by age, years of smoking, daily cigarette consumption, total smoking history, and subcutaneous adipose tissue thickness.

Variable	Meniscal injury		*P*
	Positive^*^	Negative^*^	
Age (years)	44 ± 19.70	36 ± 19.53	0.001
Years of smoking	13 ± 1.50	4 ± 4.4	0.125
Daily cigarette consumption (packs/ day)	1 ± 1.2	1 ± 1.1	0.833
Total smoking history (pack-years)	10 ± 1.64	4 ± 4.4	0.167
Subcutaneous adipose tissue thickness (mm)	23 ± 10.56	24 ± 24.41	0.900

* Values expressed as mean ± standard deviation.

The smokers had less subcutaneous adipose tissue than did the nonsmokers, and the frequency of smoking was higher in the men (*p* < 0.001 for both). Of the 30 individuals with grade 1 injuries, six (20.0%) were smokers, compared with 18 (58.1%) of the 31 individuals with grade 3 injuries. As can be seen in Table 3, smoking correlated positively with the degree of meniscal injury (*p* < 0.009).

**Table 3:** Comparison between smokers and nonsmokers, by age, sex, injury grade, and subcutaneous adipose tissue thickness.

Variable	Smoking status		*P*
	Positive	Negative	
Age (years), median (range)	37.5 (19-70)	45.0 (19-66)	0.265
Sex, n (%)			< 0.001
Male	38 (53.5)	36 (46.5)	
Female	10 (11.8)	36 (88.2)	
Injury grade, n (%)			0.009
0	1 (3.0)	32 (97.0)	
1	6 (20.0)	24 (80.0)	
2	23 (37.1)	39 (62.9)	
3	18 (58.1)	13 (41.9)	
Subcutaneous adipose tissue			
thickness (mm), median (range)	26 (10-45)	19 (10-42)	< 0.001

**Figure 4:**
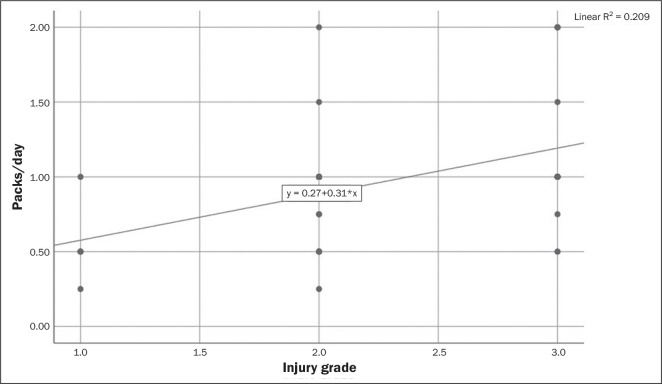
Correlation between daily cigarette consumption and meniscal injury grade.

**Figure 5:**
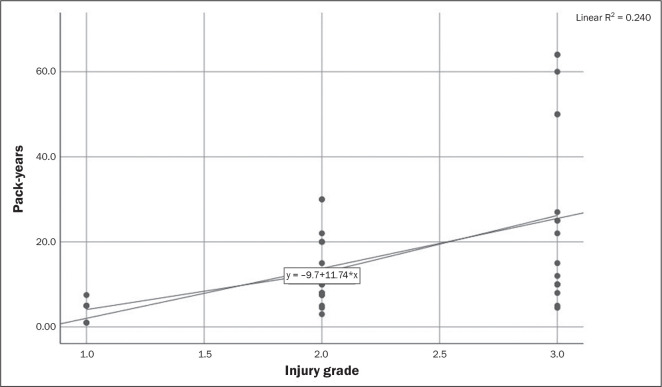
Correlation between smoking history and meniscal injury grade.

A moderate but statistically significant correlation was observed between packs/day and the injury grade as well as between pack-years and the injury grade (r = 0.462, *p* = 0.001 and r = 0.523, *p* = 0.001, respectively), as shown in Table 4, Figure 4, and Figure 5.

**Table 4:** Correlations between the variables and meniscal injury grade.

Variable	Correlation with injury grade		
	r^*^	*P*	Eta^†^
Years of smoking	0.312	0.033	0.653
Daily cigarette consumption (packs/day)	0.462	0.001	0.499
Total smoking history (pack-years)	0.523	0.001	0.764
Pack-years + subcutaneous adipose tissue thickness	0.001	0.996	-

* Spearman’s correlation coefficient.

† Point-biserial correlation coefficient.

Table 5 shows the results of the logistic regression analysis performed to evaluate the effect that certain characteristics have on the risk of meniscal injury. Smoking and age were found to increase the risk of such injury significantly, by 31.221 times (*p* = 0.001) and 1.076 times (*p* < 0.001), respectively.

**Table 5:** Logistic regression analysis of the effects of certain risk factors on meniscal injury.

Factor	B coefficient	Standard deviation	Wald	Odds ratio	*P*	95% Confidence interval
Female (reference: male)	0.294	0.595	0.245	1.342	0.621	0.418-4.307
Smoker (reference: nonsmoker)	3.441	1.074	10.263	31.221	0.001	3.803-256.302
Age	0.074	0.021	12.151	1.076	< 0.001	1.033-1.122
Subcutaneous adipose tissue thickness	-0.016	0.035	0.195	0.985	0.659	0.919-1.055
Constant	-0.001	1.001	0.000	0.999	0.999	-

## DISCUSSION

The most important finding of the present study is that smoking correlated with meniscal injury. The fact that this relationship was identified despite the lower subcutaneous adipose tissue thickness in the smoker group makes the study even more valuable.

Injuries to the meniscus result in significant musculoskeletal morbidity. The complex internal structure of the meniscus complicates treatment and meniscus repair for clinicians. The complex structure of the meniscus includes a dense extracellular matrix, proteoglycans, noncollagenous proteins, and glycoproteins. The extracellular matrix consists of 72% water and 22% collagen, interposed between cells. Meniscal cells are responsible for the synthesis and sustainability of the extracellular matrix, which determines the key properties of meniscal tissue^(1)^.

Smoking is known to be associated with many common diseases^(16)^ such as malignancies, cardiovascular diseases, lung diseases, stroke, and rheumatoid arthritis. However, the data regarding its impact on cartilage and osteoarthritis are contradictory.

In a study of 243 patients with meniscal tears, Baker et al. found no correlation between the incidence of meniscal tears and smoking^(17)^. Similarly, Zabrzyński et al.^(18)^ found that the years of smoking, packs per day, and pack-years did not correlate with functional outcomes in a sample of 50 patients with a traumatic tear of the medial meniscus. In the present study, we did not differentiate between medial and lateral meniscus injuries, and the difference between their findings and ours could be explained by the fact that our sample was three times larger. In other studies, it has been reported that smoking can impede healing from meniscal repair^(13)^ and may cause surgical repair failure^(19)^. Our sample also did not include any individuals who had undergone knee surgery. The relationship between smoking and meniscal injury could be further clarified in studies involving larger numbers of individuals, including those with a history of surgery and athletes. In addition, preoperative and postoperative evaluations of patients with meniscal injury could clarify the relationship between smoking and meniscal healing.

It is known that the components of tobacco smoke have a detrimental effect on chondrocyte function, as well as disrupting cell proliferation and extracellular matrix synthesis^(9)^. Therefore, smoking may have negative effects on the function of the chondrocytes in the knee joint cartilage and the meniscus, resulting in serious damage to the joint and meniscus.

Our findings are consistent with those of studies investigating the relationship between cartilage damage and smoking, in which it was concluded that smoking results in osteoarthritis of the knee joint^(8-12)^. However, there have also been studies indicating that smoking creates a protective effect against cartilage damage and osteoarthritis^(4-7)^. Ding et al.^(8)^ investigated the effects of smoking on cartilage volume and cartilage loss in patients with a family history of severe osteoarthritis. The authors found that smoking caused cartilage loss and defect, suggesting that gene-environment interactions play a role in the development of osteoarthritis. In a study published in 2013, Sharma et al.^(10)^ investigated the relationship between smoking and degenerative disease of the lumbar spine, showing that smoking was a major risk factor for such degeneration. In a prospective study of middle-aged adults, Davies-Tuck et al.^**(11**)^ reported that smoking had a detrimental effect on joint cartilage. In an earlier study of individuals with knee osteoarthritis, published in 2007, Amin et al.^(9)^ reported that the amount of cartilage loss at the medial tibiofemoral joint and patellofemoral joint was greater in smokers than in nonsmokers. Those authors also showed that, among the patients with symptomatic osteoarthritis at 30 months of follow-up, the smokers had more knee pain than did the nonsmokers. In a similar, two-year study of patients without knee osteoarthritis, smoking was found to increase medial and lateral cartilage loss^(12)^.

Gullahorn et al.^(7)^ investigated the effects of nicotine on glycosaminoglycan and collagen synthesis in chondrocytes. The authors found a negative relationship between smoking and osteoarthritis, suggesting that nicotine activates glycosaminoglycan and collagen synthesis in chondrocytes. In a study investigating the relationship between smoking and osteoarthritis in China, Zhang et al.^(5)^ observed a negative correlation between the two. In a study published in 2016, Kong et al.^(6)^ also found an inverse relationship between smoking and osteoarthritis, that relationship being more pronounced in men than in women. Sandmark et al.^(4)^ showed that smoking decreased the risk of osteoarthritis and that being overweight increased that risk, especially in women. Ford et al.^(2)^ found an important relationship between body mass index and meniscal tears that required surgery.

In contrast with some studies in the literature^(2,4)^, we did not find a significant relationship between being overweight and having a meniscal injury. Although we measured the thickness of subcutaneous adipose tissue in the lower extremity in our weight assessment, we think that there is a need for a further studies, evaluating central and peripheral obesity separately, in order to investigate this contradiction.

It has been reported that some structural abnormalities, such as discoid meniscus, ligamentous laxity, and biconcave tibial plateau, increase the risk of meniscal injury^(20)^. In the present study, structural abnormalities were not included as variables, which limits the study. In future studies, the effect of these abnormalities together with smoking could be highlighted. Other limitations of our study include the small size of the study population. Prospective studies with more participants could obtain more meaningful results. Another potential limitation is the fact that we did not evaluate the relationships that other parameters of degeneration on radiography have with meniscal injury. In addition, individuals who regularly participated in sports were excluded from our study because of the high probability of meniscal injury among such individuals. The use of subcutaneous adipose tissue thickness, rather than body mass index, as an indicator of obesity was also a limitation of the study. Furthermore, the MRI findings were assessed by two radiologists establishing consensus, so interobserver reliability was not assessed.

In conclusion, smoking is a major socioeconomic and public health problem worldwide. The results of our study indicate that smoking is positively associated with the development and degree of meniscal injury.

## References

[1] Fox AJS, Bedi A, Rodeo SA. (2012). The basic science of human knee menisci: structure, composition,
and function. Sports Health.

[2] Ford GM, Hegmann KT, White Jr GL (2005). Associations of body mass index with meniscal
tears. Am J Prev Med.

[3] Snoeker BAM, Bakker EWP, Kegel CAT (2013). Risk factors for meniscal tears: a systematic review including
meta-analysis. J Orthop Sports Phys Ther.

[4] Sandmark H, Hogstedt C, Lewold S (1999). Osteoarthrosis of the knee in men and women in association with
overweight, smoking, and hormone therapy. Ann Rheum Dis.

[5] Zhang Y, Zeng C, Li H (2015). Relationship between cigarette smoking and radiographic knee
osteoarthritis in Chinese population: a cross-sectional
study. Rheumatol Int.

[6] Kong L, Wang L, Meng F (2017). Association between smoking and risk of knee osteoarthritis: a
systematic review and meta-analysis. Osteoarthritis Cartilage.

[7] Gullahorn L, Lippiello L, Karpman R. (2005). Smoking and osteoarthritis: differential effect of nicotine on
human chondrocyte glycosaminoglycan and collagen synthesis. Osteoarthritis Cartilage.

[8] Ding C, Cicuttini F, Blizzard L (2007). Smoking interacts with family history with regard to change in
knee cartilage volume and cartilage defect development. Arthritis Rheum.

[9] Amin S, Niu J, Guermazi A (2007). Cigarette smoking and the risk for cartilage loss and knee pain
in men with knee osteoarthritis. Ann Rheum Dis.

[10] Sharma MK, Petrukhina E. (2013). Strong association of smoking with lumbar degenerative spine
disease. The Open Neurosurgery Journal.

[11] Davies-Tuck ML, Wluka AE, Forbes A (2009). Smoking is associated with increased cartilage loss and
persistence of bone marrow lesions over 2 years in community-based
individuals. Rheumatology (Oxford).

[12] Ding C, Martel-Pelletier J, Pelletier JP (2008). Two-year prospective longitudinal study exploring the factors
associated with change in femoral cartilage volume in a cohort largely
without knee radiographic osteoarthritis. Osteoarthritis Cartilage.

[13] Ruzbarsky JJ, Maak TG, Rodeo SA., Miller MD, Thompson SR (2020). DeLee, Drez and Miller’s orthopaedic sports medicine.

[14] Kruger J, Ham SA, Kohl 3rd HW (2007). Characteristics of a “weekend warrior”: results from two national
surveys. Med Sci Sports Exerc.

[15] Li CA, Kim MK, Kim IH (2013). Correlation of histological examination of meniscus with MR
images: focused on high signal intensity of the meniscus not caused by
definite meniscal tear and impact on MR diagnosis of tears. Korean J Radiol.

[16] Edwards R. (2004). The problem of tobacco smoking. BMJ.

[17] Baker P, Coggon D, Reading I (2002). Sports injury, occupational physical activity, joint laxity, and
meniscal damage. J Rheumatol.

[18] Zabrzyński J, Paczesny Ł, Zabrzyńska A (2022). Smoking has no influence on outcomes after repair of the medial
meniscus in the hypo and avascular zones-a pilot study. Int J Environ Res Public Health.

[19] Blackwell R, Schmitt LC, Flanigan DC (2016). Smoking increases the risk of early meniscus repair
failure. Knee Surg Sports Traumatol Arthrosc.

[20] Adams BG, Houston MN, Cameron KL. (2021). The epidemiology of meniscus injury. Sports Med Arthrosc Rev.

